# A Hierarchical Building Segmentation in Digital Surface Models for 3D Reconstruction

**DOI:** 10.3390/s17020222

**Published:** 2017-01-24

**Authors:** Yiming Yan, Fengjiao Gao, Shupei Deng, Nan Su

**Affiliations:** 1 Institute of Information Technology, Harbin Engineering University, Harbin 150001, China; 2 Institute of Automation of Heilongjiang Academy of Sciences, Harbin 150001, China; 3 Department of Information Engineering, Harbin Institute of Technology, Harbin 150001, China; dengshupei2016@126.com (S.D.); sunan_hit@hotmail.com (N.S.)

**Keywords:** building segmentation, digital surface model, remote sensing, 3D reconstruction

## Abstract

In this study, a hierarchical method for segmenting buildings in a digital surface model (DSM), which is used in a novel framework for 3D reconstruction, is proposed. Most 3D reconstructions of buildings are model-based. However, the limitations of these methods are overreliance on completeness of the offline-constructed models of buildings, and the completeness is not easily guaranteed since in modern cities buildings can be of a variety of types. Therefore, a model-free framework using high precision DSM and texture-images buildings was introduced. There are two key problems with this framework. The first one is how to accurately extract the buildings from the DSM. Most segmentation methods are limited by either the terrain factors or the difficult choice of parameter-settings. A level-set method are employed to roughly find the building regions in the DSM, and then a recently proposed ‘occlusions of random textures model’ are used to enhance the local segmentation of the buildings. The second problem is how to generate the facades of buildings. Synergizing with the corresponding texture-images, we propose a roof-contour guided interpolation of building facades. The 3D reconstruction results achieved by airborne-like images and satellites are compared. Experiments show that the segmentation method has good performance, and 3D reconstruction is easily performed by our framework, and better visualization results can be obtained by airborne-like images, which can be further replaced by UAV images.

## 1. Introduction

For making 3D city maps and visualization [1,2,3], 3D reconstruction of buildings using remote sensing (RS) images and digital surface models (DSMs), is always a complicated but necessary work. To better generate digital building models, 3D reconstruction of buildings using DSMs has been widely investigated. The main task is to reconstruct all the outer surfaces of a building, which are composed by the roof and all facades.

With the development of airborne light detection and ranging (LiDAR), high accuracy DSM can be obtained after pre-processing the original LiDAR data. 3D building reconstructions using DSMs are generally based on models [4,5,6]. A number of types of 3D models of buildings are firstly constructed offline, then a matched one for each segmented buildings in the DSM is used for the 3D reconstruction. A limitation of these methods is the over-reliance on the completeness of the offline-constructed models of the buildings, which cannot be easily guaranteed since in modern cities there are a variety of types of buildings. Moreover, 3D modeling and model selection are labor-intensive work, in which complex interactive manual operations are always needed. Furthermore, the facades of buildings are not properly processed in most of these methods for further texture-mapping and visualization.

By using high accuracy DSM and texture-images of the roofs and facades of the buildings, 3D reconstruction can be performed without creating offline models [7,8,9], but most of these kinds of model-free methods only focus on reconstruction of the roofs of buildings, or do not process the texture-images. In our idea, a DSM and the texture-images of the outer-surfaces of buildings can be associated for a better reconstruction. Firstly the shapes of the roofs of buildings need to be extracted and analyzed.

DSMs can mostly be processed as a digital image. Various image segmentation methods are employed [10,11,12] for DSM processing, such as classification, edge detection, and some other filter-based methods. Active contour-based methods are also used. For instants, a level-set is suitable for efficiently searching the contours of buildings in DSMs, since buildings have larger values than other objects on the ground. However, roof details cannot be detected if the initialization is not properly done. Moreover, when a high tree is next to a building, good performance on the edges of the adjacent building generally cannot be obtained by level-set approaches. Jia et al. [13] proposed a level-set-based method for building modeling, but it is only applicable to isolated buildings in relatively flat areas. The processing of details on the edges needs to be improved. Iovan et al. [14] proposed a support vector machine (SVM)-based tree extraction method in DSMs with prior knowledge. This could be expanded for further building segmentation since only trees and buildings are higher. Similarly, a supervised classification method was introduced for change-detection of buildings [15]. Buildings are segmented by height, spectra, and shape information from a DSM, assisted by orthophotos of the same area. However, prior knowledge for supervised classification is not easily obtained, and it always determines the segmentation performance. Liu et al. [16] employed an urban DSM segmentation by extended locally excitatory globally inhibitory oscillator networks (LEGION), which is an unsupervised classification-based method without prior knowledge, but it fails to do small features and boundaries of complex buildings. McCann and his team summarized the limitations of various generic image segmentation methods and engineered systems. They concluded that most existing segmentation methods are not successful on histopathology images, since these methods vary in how the segmentation regions are parameterized and whether edge or region information is used [17]. In consideration of that, McCann modeled images as occlusions of random texture for segmentation (ORTSEG), and proposed a new unsupervised mathematical framework method based on local histograms. The method handles the difficult class of edgeless images and has excellent applicability to histology image segmentation and it could also be expanded for building segmentation in DSM, and thus improve the performance on edges, but the limitations of this method are that certain numbers of parameters need to be properly set for different conditions, and any overall terrain undulation could have a negative impact on the class judgments in certain local regions near the building.

Considering both efficient detection and more accurate segmentation on the edges, we introduce a hierarchical building segmentation method. Rough positioning of buildings is performed by level-set in a global DSM first, and then all regions of buildings are processed separately by the ORTSEG method to decrease negative terrain factors, and improve the segmentation accuracy.

Once the roof of a building is extracted from the DSM, only the 3D structure of the roof is obtained, but all facades of the building need to be generated. Since roof contours might be of different height, the part of the facade which is close to the roof is uneven. As a result, certain criteria must be defined referring to the range of each facade. A roof-contour and texture-image guided interpolation (RTGI) method is introduced for facade generation and texture-mapping of buildings by synergizing with texture-images of each outer-surface of the building, which are collected from RS optical images of different views of the building obtained by satellite or unmanned aerial vehicles (UAVs). UAVs are currently performing significant roles for city remote sensing missions, and higher resolution texture-images could be collected by UAVs. After pasting these texture-images onto the corresponding facade generated by RTGI, the visualization results could be greatly improved. Furthermore, UAVs equipped with LIDAR can collect high precision DSMs efficiently in the near future, and better use of our method will be made. The main contributions of this study are:

(1) A novel 3D reconstruction framework without offline models is proposed, and the introduced RTGI could effectively help generate facades of a building. Our framework could reconstruct 3D buildings with limited RS resources, and reduce complicated manfully modeling work.
(2) Based on the level-set and ORTSEG techniques, we propose a hierarchical segmentation method for extracting buildings from DSMs, named LS-ORTSEG. It can greatly reduce the negative factors that result from the uncertain terrain and the limitations of the different segmentation algorithms.

This paper is organized as following: the model free framework for 3D reconstruction is introduced in Section 2. The hierarchical segmentation method LS-ORTSEG is discussed in Section 3, then Section 4 presents our analysis and experimental results. Section 5 concludes this work, and discusses the advantages, limitations and future work of this method.

## 2. Framework of 3D Reconstruction

**Figure 1:**
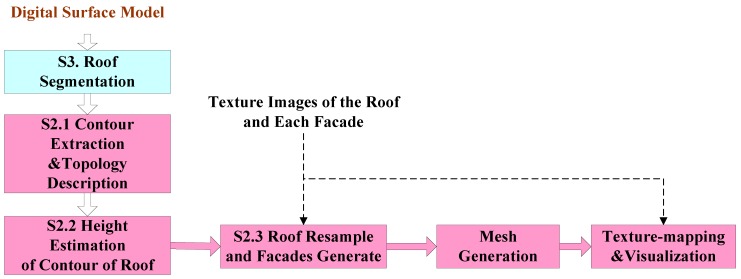
Flowchart of 3D building reconstruction and visualization.

As shown in Figure 1, the first step is to segment the roof of buildings from DSM. Details are discussed in Section 3.

Then, the contour of the roofs are extracted and analyzed, and the numbers of facades are found and then coded. After that, the actual heights of all sampled points of the contour are estimated by comparing the elevations between inner points and outer points. Further, the RTGI method is introduced for generating all the outer-surfaces of the building, guided by the coded number of facades and sizes of different texture-images, and then the 3D points-sets of each outer-surface are obtained. We then gather the 3D points-sets of all the outer-surfaces of the buildings, and generate the mesh. Finally, the texture images are mapped onto the corresponding triangle-area of each outer-surface, and we visualize the mapping result.

### 2.1. Extraction and Coding of Roof Edge-Lines

**Figure 2:**
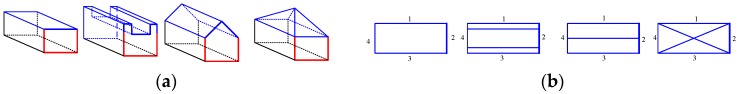
Examples of four types of buildings. (**a**) The four buildings; (**b**) Edge-lines of the roofs.

**Figure 3:**
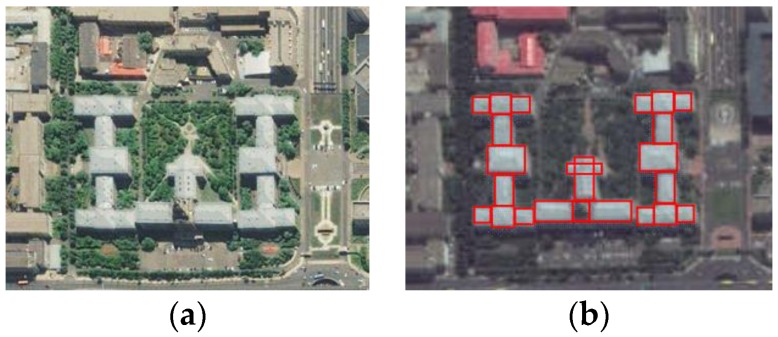
Blocks of an irregular top building. (**a**) An irregular top building; (**b**) Divided blocks.

Since the edge-line of the roof is the intersection of each facade and the roof, it is important to generate each facade. In DSM data, like in orthographic images, buildings are represented by quantified grids (like pixels) in the horizontal direction, and only the elevation of the blue part of a facade can be obtained, as shown in Figure 2a. When the roof of a building is extracted from the DSM, Su’s [18] and Qiu’s [19] work are taken for optimizing the extraction results and further determine the topology of the building. Then buildings with irregular top shapes can be divided into several blocks, as shown in Figure 3. The main steps are as follows: (1) line extraction methods like LSD [20], can be used to find the line features of the building; (2) after removing some erroneous lines caused by angle and length-based restrictions, the remaining lines can form a group of parallelogram grids, which divide the irregular shape of roof into different regions; (3) a specific decision criterion is used to judge each region as building or non-building class, considering both the segmented result and the restriction of the parallelogram grids. All the blocks can be coded in a customized order, and then the edge-lines of the roof of each block or a simple shaped building can be directly coded in a clockwise or counterclockwise, as shown in Figure 2b.

### 2.2. Height Estimation of Edge-Lines

After extracting and coding the roof edge-lines, the heights of the edge-lines need to be estimated for further facade generation. In DSM, only the elevation of buildings is directly obtained, but the heights of buildings need to be estimated. Here we estimate them by comparing the elevation of the roof-contour and foot-contour. Mathematical morphology methods are used for extracting the roof-contour *RC* by an erosion operation after a dilation operation, and the foot-contour *FC* by a dilation operation after an erosion operation. If *n* and *m* are respectively the numbers of points of the *RC* and *FC*, the height of each point of the *RC* is estimated by Equation (1):

(1) $$H(i)=RC(i)-\frac{1}{3}\sum_{m=1}^{3}FC_{c3}(m)\;i=1,2,...,n$$

**Figure 4:**
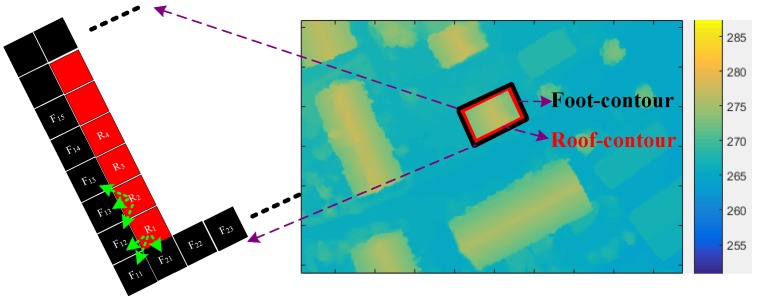
Height estimation.

As shown in Figure 4, *RC*(*i*) is elevation of the *i*-th point of the *RC*, and *FC_c3_*(*m*) is the elevation of the *m*-th point of the closest three points around the *i*-th point of the *RC*.

### 2.3. Roof-Contour and Texture-Image-Guided Interpolation

**Figure 5:**
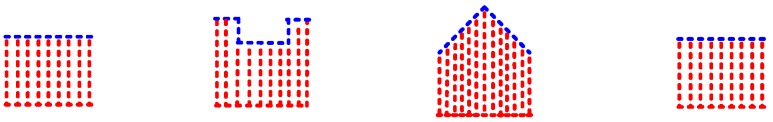
Generated building facades with interpolated points.

Since each edge-line point has the same horizontal coordinate as its corresponding part on the facade below, only the ‘Z-coordinate’ needs to be interpolated. Assuming the size of a texture image is *M* × *N*, then the facade can be interpolated as shown in Figure 5. Firstly, we resample the edge-line to fit the image with the nearest neighbor criterion, and the new number of points of the *i*-th edge-line *P*’ will be *N*. Then we interpolate each row *n*’ (*n*’ = 1, 2, ..., *P*’) of the side from the foot to the top by Equation (2):

(2) $$S_{i}^{n\prime }(p)=\frac{H_{i}(n\prime )}{M}\cdot p\;p=1,2,...,M$$

After finishing interpolating, each facade can be generated by a 3D point-set as seen in Figure 5, and then 3D meshes are generated by the combined point-sets including all outer-surfaces for further detailed texture-mapping. The texture-mapping error $\tilde{E}$ could be evaluated by Equation (3), where we select *N_T_* testing points, and compare the mean error of the 3D coordinates between the testing points ($p_{x}^{t}(i)$, $p_{y}^{t}(i)$, $p_{z}^{t}(i)$) and the corresponding measured points ($p_{x}^{m}(i)$, $p_{y}^{m}(i)$, $p_{z}^{m}(i)$):

(3) $$\tilde{E}=\frac{1}{N_{T}}\sum_{i=1}^{N_{T}}\sqrt{{\left(p_{x}^{t}(i)-p_{x}^{m}(i)\right)}^{2}+{\left(p_{y}^{t}(i)-p_{y}^{m}(i)\right)}^{2}+{\left(p_{z}^{t}(i)-p_{z}^{m}(i)\right)}^{2}}$$

## 3. A Hierarchical Building Segmentation Based on the LS-ORTSEG Method

**Figure 6:**
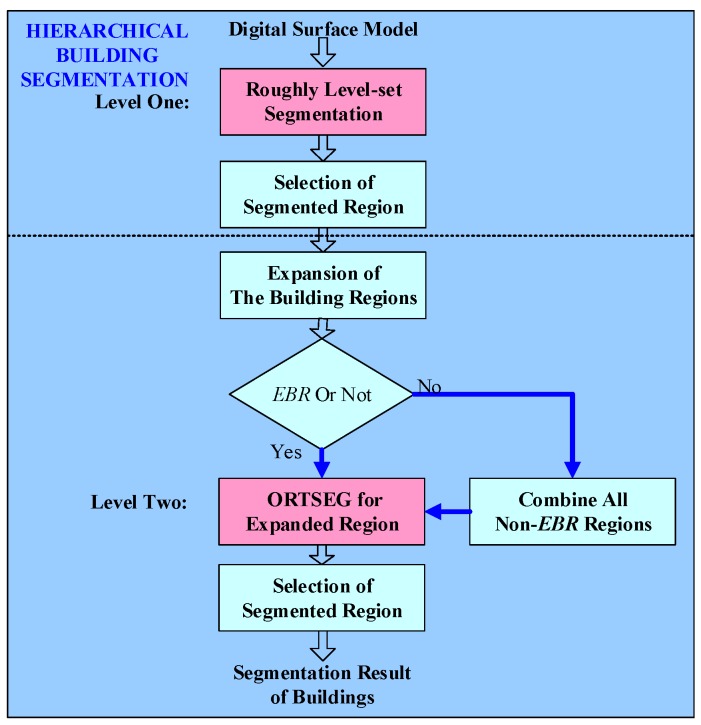
Hierarchical LS-ORTSEG building segmentation.

As illustrated above, building segmentation is significant to our 3D reconstruction framework. Since DSMs used for better 3D reconstruction have quite high resolution, serrated building boundaries can be happen in the DSM, and also branches and leaves of trees around the building might cause bad boundaries. As a result, the edges and details of the roof need to be improved when segmenting. We consider the advantages of the level-set and ORTSEG method, and hierarchically segment the DSM with following steps, shown in Figure 6.

**Figure 7:**
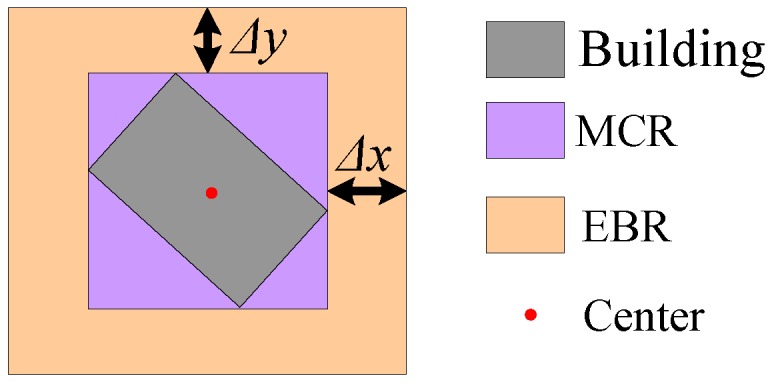
Region expansion for level-two segmentation.

**Figure 8:**
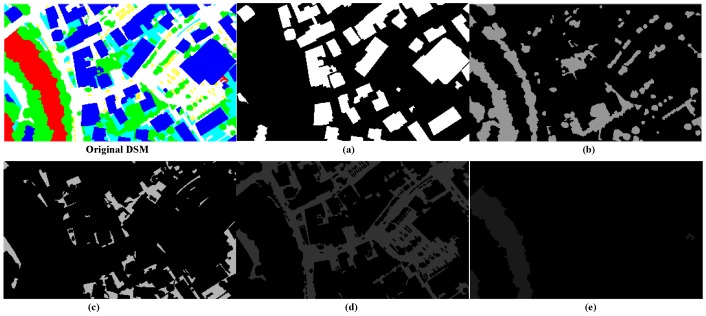
Modeling a DSM as an occlusion of textures. (**a**–**e**) is one of the five random textures.

(1) A classic Chan-Vese model-based level-set is introduced for level-one segmentation of the DSM, using a circle mask in the whole DSM. Then the *N_ls_* reigions are roughly segmented as buildings.
(2) Restrict building regions (*BR*) adopting some certain geometric properties. Here, we set a threshold *TH_Area_* for the area of the segmented region as Equation (4):
  (4) $$Area\underset{i=1,2,..,N_{ls}}{\left\{i\right\}}=NUM_{pixel}\left\{i\right\}\cdot res_{DSM}<TH_{Area}$$
  where *NUM_pixel_* is the number of pixels in the region and *res_DSM_* is the DSM resolution. Then *N_ssr_* regions are identified as buildings:
(3) Extract the minimum circumscribed rectangle (*MCR*) of each building region. As shown in Figure 7, the *N_EBR_* expanded building regions (*EBR*) are obtained by Equation (5):
  (5) $$EBR_{i}=MCR_{i}\cup EX_{i}\left(\Delta x,\Delta y\right)$$
  where the *i*-th *EBR* is the union of *i*-th *MCR* and *EX* in the original DSM region and *Δx*, *Δy* are the expansion distance for the horizontal and vertical directions. Note that when a building is near the boundary of the input DSM, the corresponding side of *Δx* or *Δy* must be decreased to fit the boundary, and the other side retains the original value. After obtaining all the *EBR*s, we combine all non-*EBR*s as background, by setting the values of all the *EBR*s in the original DSM to a large enough constant, and the segmented results of these positions will be replaced by that of *EBR*s.
(4) Then, level-two segmentation is performed on both all *EBR*s and the background by the ORTSEG method. The labeling function γ of an image, which defines the class of each pixel with different numbers, splits the image into *N_ort_* regions. That models an image as occlusions of random textures $I=O_{\gamma }{\left\{I_{n}\right\}}_{n=0}^{N_{ort}-1}$ (Figure 8).

McCanna [17] proved that a local histogram could be approximated by a convex combination of the value histograms of those regions. As a result, segmentation is handled as an optimization, which is to search the labeling function γ, for the best approximation of the local histograms *L_ω_H* by the statistical function *h*_{*n*}_*(r)* and probability density function ${\hat{p}}_{h}^{Xn}$ of the *N_ort_* regions:

(6) $$\underset{\gamma }{\mathrm{argmin}}\left\|L_{\omega }H-\sum_{n=0}^{N_{ORT}-1}[\omega *h_{\left\{n\right\}}(\gamma )]{\hat{p}}_{h}^{Xn}\right\|$$

**Figure 9:**
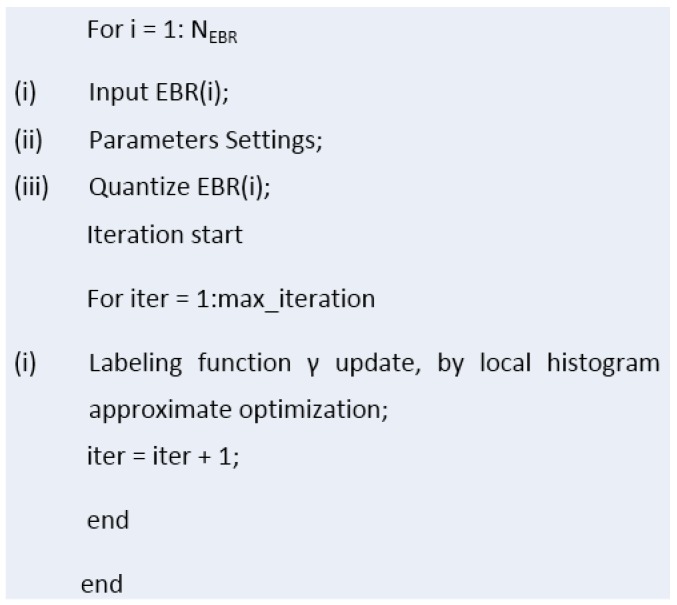
Main flow of LS-ORTSEG based *EBR*s segmentation.

Similarly, DSM can be segmented using ORTSEG. However, since the labeling function γ of ORTSEG is to fit global statistical properties, if ORTSEG were used on the whole DSM, the potential overall undulating terrain might lead to poor performance in the local regions of different buildings. For example, buildings on the mountainside and on the foot could be identified as different labels. As a result, we intend to improve the local accuracy by segmenting all the *EBR*s and the background instead. The main flow of LS-ORTSEG based *EBR*s segmentation is as shown in Figure 9.

## 4. Experimental Section

### 4.1. Data Statement

**Figure 10:**
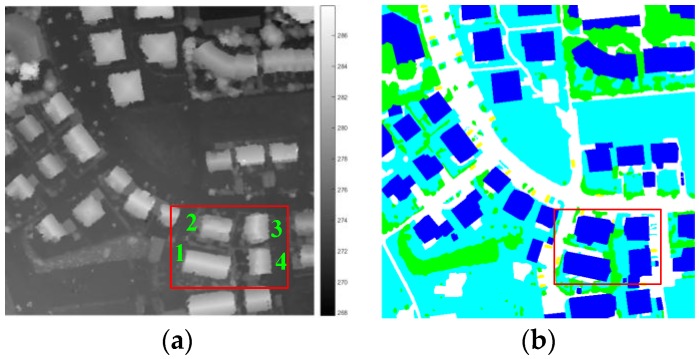
Dataset 1: (**a**) original DSM shown as image, and 1, 2, 3 and 4 is the serial number of the four buildings; (**b**) ground truths.

Three groups of datasets are collected for our experiments. As shown in Table 1, Dataset 1 was captured by an aircraft over Vaihingen in Germany at a height of 500 m, which can be reached by UAVs. The dataset is collected for segmentation with ground truths, as shown in Figure 10. The Vaihingen dataset was provided by the German Society for Photogrammetry, Remote Sensing and Geoinformation (DGPF) [21]. The DSMs of Datasets 2 and 3 were obtained from the OpenTopography website [22], and the texture-images of the two datasets are from satellites and Google-Earth, for testing the 3D reconstruction performance.

### 4.2. Building Segmentation Using Hierarchical Method

After investigating the terrain of this scene using the actual map and the DSM data, four adjacent buildings were selected to verify the reliability and robustness of our method. The reasons were as follows: (1) they are adjoined, and the surroundings are similar; (2) their terrain factors are similar but have certain distintions, especially between Buildings 1 and 2, and also between Buildings 3 and 4; (3) they have several kinds of shapes: Buildings 3 and 4 are similar but different from the others; (4) they have different orientations, and DSMs have different degrees of error in different directions.

Level-set, ORTSEG, and LS-ORTSEG are used for segmenting these regions. In ORTSEG, besides the quantize and deconvolution methods that need to be selected, many other parameters need to be set. ‘Hist.wSize’ (sets the size of the histogram filter) and ‘NumTextures’ (sets the number of textures to segment to) are the key parameters that affect the segmentation results. ‘Hist.wSize’ is generally set according to resolution and experience. ‘NumTextures’ is set to 5 for ORTSEG, since the number of classes is 5 in the ground truth of Dataset 1. Generally, better results should be obtained if we set ‘NumTextures’ close to the number of classes in the ground truths. However, in LS-ORTSEG, ‘NumTextures’ are strictly set to 2 since most buildings in these *EBR*s are simple shapedones. Other uniform settings of ORTSEG and LS-ORTSEG are as follows: (1) ‘quantizeMethods’ are set to ‘*k*-means’; (2) ‘quantize.numColors’ = 8; (3) factor.numReps = 25 (it decides how many times to repeat the factorization).

**Figure 11:**
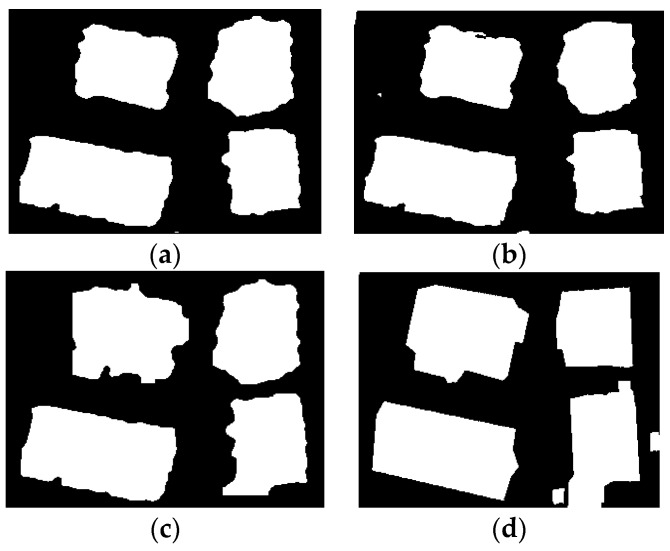
Segmentation results of four buildings by different methods. (**a**) Level-Set; (**b**) ORTSEG; (**c**) LS-ORTSEG; (**d**) Ground Truths. The ‘Hist.wSize’ is set to 3 for both ORTSEG and LS-ORTSEG.

The results obtained with these general settings are shown in Figure 11. Similar performance is obtained for Buildings 1 and 3 by the three methods, but LS-ORTSEG gets better results on Buildings 2 and 4.

**Figure 12:**
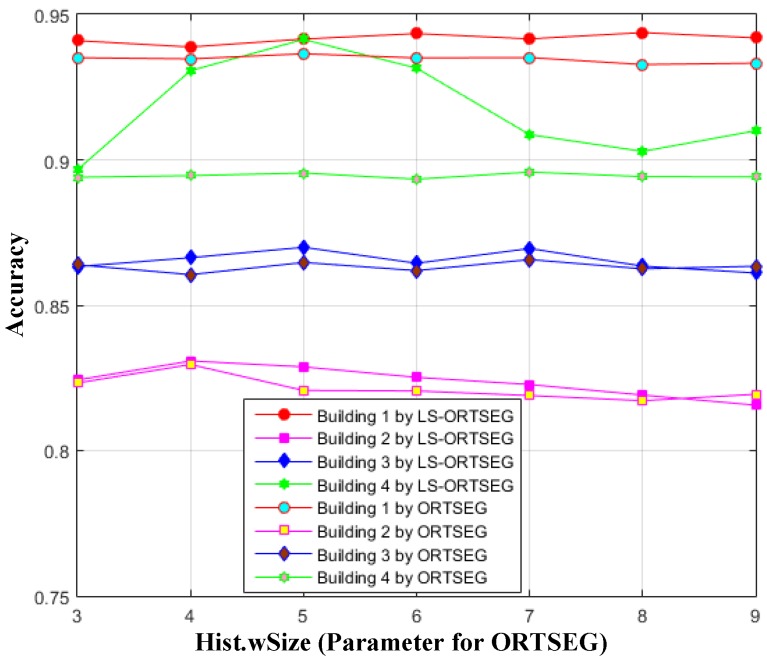
Accuracy using different window sizes. The ‘NumTextures’ are set to 5 for ORTSEG, and ‘Hist.wSize’ are set from 3 to 9 for both ORTSEG and LS-ORTSEG.

ORTSEG and LS-ORTSEG were further compared when using different parameter settings. Accuracy of segmentation is evaluated by Equation (7):

Accuracy = (TP + TN)/(TP + FN + FP + TN)(7)

where TP is the number of points of the building in the ground truth matrix. FP is the number of non-building points in the ground truth matrix. TN is the number of correctly segmented points of the building according to the ground truth matrix. FN is the number of erroneous segmented points of the building according to the ground truth matrix. Firstly, ‘NumTextures’ is fixed to 5 for ORTSEG and LS-ORTSEG, and we draw the accuracy of the two methods when ‘Hist.wSize’ are set from 3 to 9, as shown in Figure 12, which illustrates that LS-ORTSEG gives better accuracy than ORTSEG for most cases.

**Figure 13:**
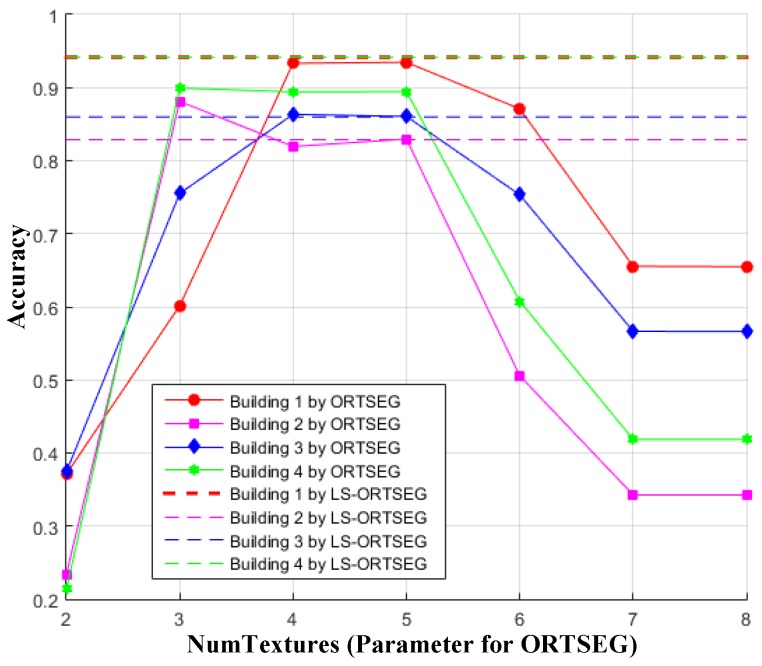
Accuracy using different texture numbers. The ‘Hist.wSize’ are set to 5, and ‘NumTextures’ are set from 2 to 8 for both ORTSEG and LS-ORTSEG.

The accuracy analysis when ‘Hist.wSize’ is fixed to 5 and ‘NumTextures’ are set from 2 to 8, is shown in Figure 13. It indicates that ‘NumTextures’ should be set close to the number of classes of the ground truths for better segmentation. Otherwise, when ‘NumTextures’ is set too small or too large, the negative impact of the terrain will dominantly destroy the segmentation, and poor accuracy is obtained.

**Figure 14:**
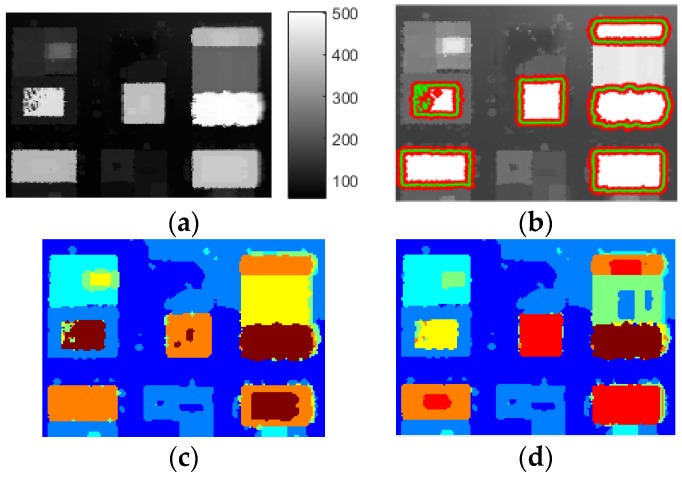
Original DSM and segmentation results of Dataset 2. (**a**) DSM (**b**) Level-set; (**c**) ORTSEG; (**d**) LS-ORTSEG. ‘Hist.wSize’ = 3, ‘NumTextures’ = 7 for both ORTSEG and LS-ORTSEG.

**Figure 15:**
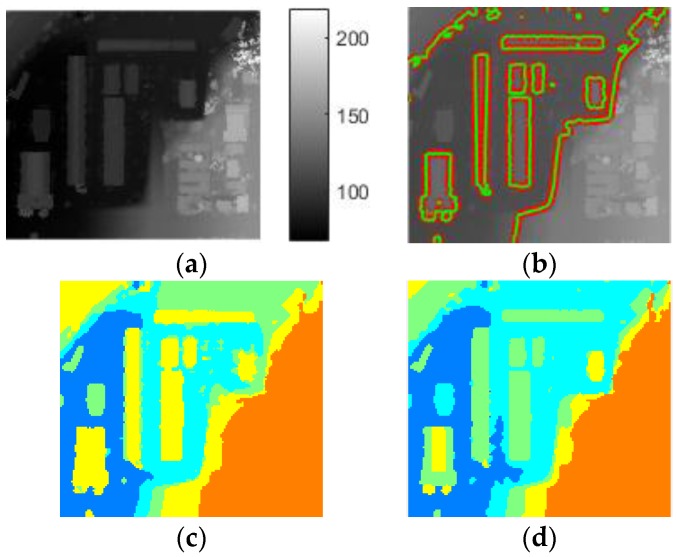
Original DSM and segmentation results of Dataset 3. (**a**) DSM (**b**) Level-set; (**c**) ORTSEG; (**d**) LS-ORTSEG. ‘Hist.wSize’ = 3, ‘NumTextures’ = 7 for both ORTSEG and LS-ORTSEG.

The best accuracy of different methods is listed in Table 2. It illustrates that LS-ORTSEG achieved better segmentation than other methods. Moreover, Datasets 2 and 3 are processed, as shown in Figure 14 and Figure 15. It could be seen that level-set is not as sensitive to the terrain, but it missed some of the buildings. ORTSEG obtained a good result while LS-ORTSEG can further segment the small prominent structures on the roofs, which is crucial to analyze the roof contour.

### 4.3. 3D Reconstruction of Buildings

**Figure 16:**
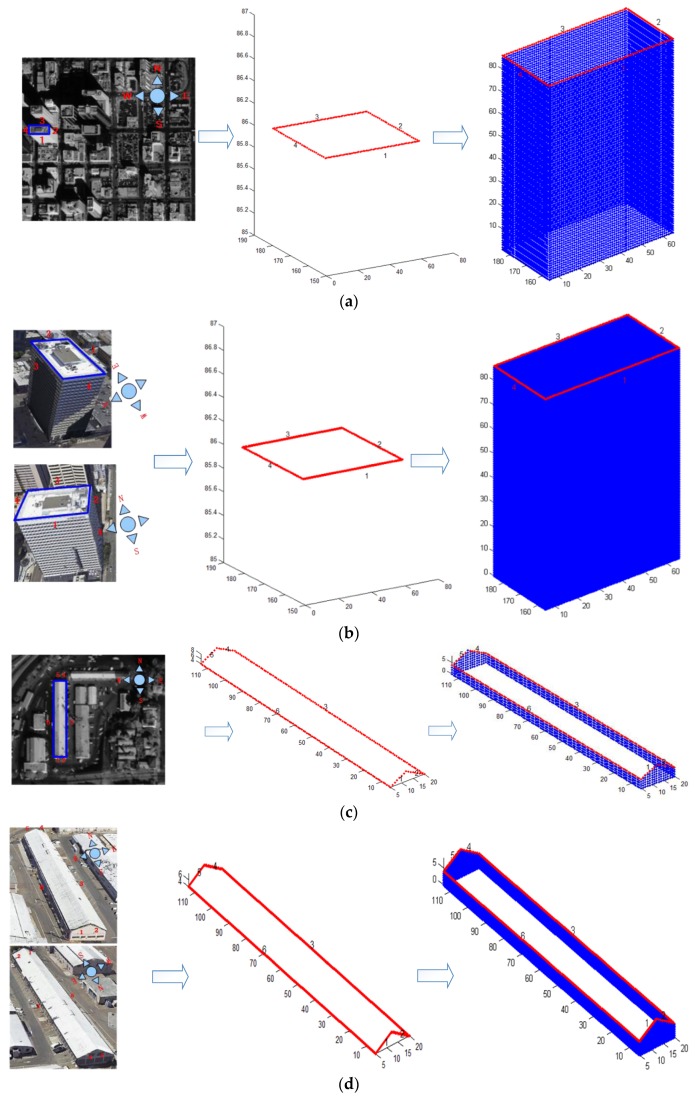
Facade generation of buildings in different cases. (**a**) Facade generation of Building A by satellite texture-images; (**b**) facade generation of Building A by airborne-like texture-images; (**c**) facade generation of Building B by satellite texture-images; (**d**) façade generation of Building B by airborne-like texture-images.

As shown in Figure 16, we selected two buildings from Datasets 2 and 3, respectively, to show the subsequent 3D reconstruction. After extracting the building roofs from the DSM, the facades are generated by RTGI.

Different texture cases were compared in the experiments. For one case, facade images of buildings captured by IKONOS are cut as texture-images. The resolutions are 1 m/pixel. For another case, facade images of the buildings are taken from Google-Earth to simulate airborne-like images, and the resolutions were resampled to 0.3 m/pixel. The texture-images can be replaced by other UAV images if collected. The heights of the facades of Building A and Building B are 86 m and 4 m, respectively. The interpolations were strictly fit to the texture-images. 3D-points were properly generated for the two buildings.

**Figure 17:**
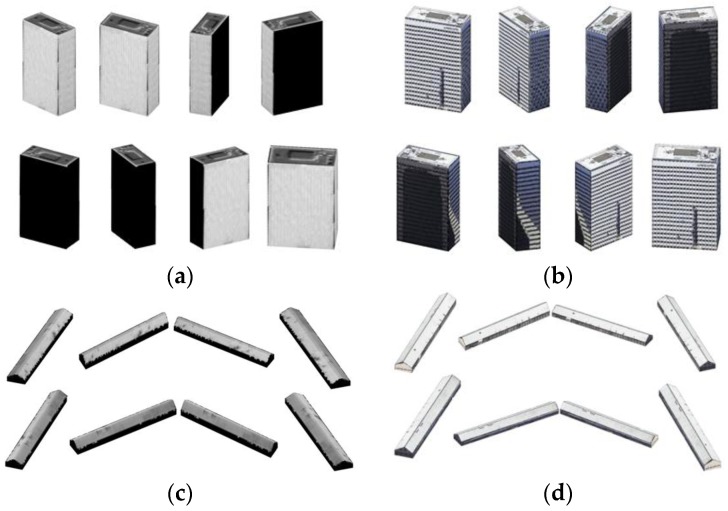
3D reconstruction of buildings in Dataset 2 and Dataset 3. (**a**) 3D reconstruction of Building A in multiple views by satellite texture-images; (**b**) 3D reconstruction of Building A in multiple views by airborne-like texture-images; (**c**) 3D reconstruction of Building B in multiple views by satellite texture-images; (**d**) 3D reconstruction of Building B in multiple views by airborne-like texture-images.

**Figure 18:**
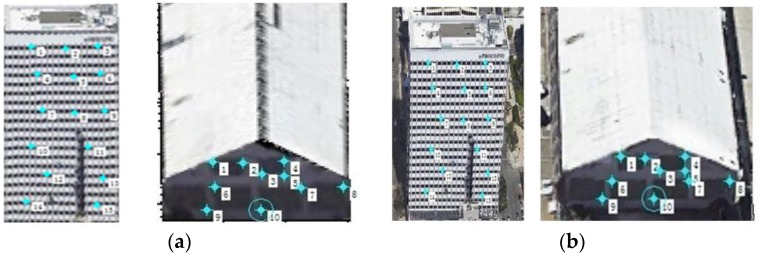
Selected points for accuracy verification. (**a**) Testing points of the two buildings; (**b**) standard points selected from Google-Earth.

3D-points of all sides of the buildings were collected to generate the triangular mesh. Then the buildings were reconstructed by the triangular mesh and all texture-images (black texture for invisible facades in different cases), as shown in Figure 17a–d. They illustrate that RTGI could process texture-images using different resolutions adaptively, and higher reconstructed quality were obtained from better texture-images. To verify the accuracy of our method, two groups of testing points are selected: 15 points for Building A, 10 points for Building B, as shown in Figure 18a,b. In the maximum display scale of the buildings in Google-Earth, the texture of the two buildings show a perfect fit and were clearly shown in the screen, so we manually selected and measured the position of the points as a standard, referring to the supervised geometric information of Google-Earth. Then we calculated the error at different directions between the testing points and the selected standard points by Equation (3). The results were acceptable, as listed in Table 3.

## 5. Discussion and Conclusions

In this paper, a hierarchical segmentation method named LS-ORTSEG was proposed to enhance the performance of a model-free framework for building 3D reconstructions with RS resources. In this framework, 3D information of buildings are segmented from DSM, and then together with texture-images, facades of the buildings are generated by the RTGI method, and texture-images are further pasted onto the corresponding outer-surfaces to finish the 3D reconstruction. LS-ORTSEG was used to decrease the negative factors resulting from the uncertain terrain.

Three groups of datasets were used, which were collected from different areas of the world. Some specific buildings were selected to test the performance of our proposed methods. In the experiments, the advantages of our LS-ORTSEG in different cases compared to traditional methods were verified by Datasets 1, 2 and 3. The optimization of parameter settings of LS-ORTSEG could be a future objective. Moreover, the 3D reconstruction performance showed the effectiveness of our framework, and an acceptable 3D reconstruction accuracy was obtained according to the measured coordinates of the testing points. Texture-images from aerial images gave better results than satellite images. Besides, 3D reconstruction results, especially for irregularly shaped roofs, can be found in other work of our team [19,23]. Furthermore, UAV images, which can take texture-images with more ideal angles and spatial resolution, are more easily collected now, making our proposed method having scalability and interesting practical prospects.

## Figures and Tables

**Table 1:** Statement of datasets.

SN of Datasets	1	2	3
Position	Vaihingen, Germany	San Diego, CA, USA	San Diego, CA, USA
DSM collection	Airborne	Airborne	Airborne
Texture-images collection	Airborne	Satellite	Satellite
Ground Truths of DSM	YES	NO	NO
Test-points of facades?	NO	By Google Earth	By Google Earth
Resolution of DSM	0.09 m	1.0 m	1.0 m
Resolution of Texture-images	-	1.0 m/0.3 m	1.0 m/0.3 m

**Table 2:** Accuracy of different methods.

Method	Accuracy			
	Building 1	Building 2	Building 3	Building 4
Level Set	92.26%	82.44%	84.57%	90.27%
ORTSEG	93.64%	82.96%	86.57%	89.58%
LS-ORTSEG	94.37%	83.08%	86.99%	94.14%

**Table 3:** Reconstruction errors of testing points.

Serial Number of the Testing Points	Building A	Building B
1	0.1	0.4
2	0.9	0.1
3	0.4	0.6
4	0.8	0.0
5	1.6	0.3
6	0.8	0.4
7	0.3	0.4
8	1.1	0.5
9	0.2	0.3
10	0.8	0.2
11	1.1	-
12	0.2	-
13	0.7	-
14	0.6	-
15	1.1	-
Mean Error	0.7	0.3
